# Insight into the Topological Nodal Line Metal YB_2_ with Large Linear Energy Range: A First-Principles Study

**DOI:** 10.3390/ma13173841

**Published:** 2020-08-31

**Authors:** Yang Li, Jihong Xia, Rabah Khenata, Minquan Kuang

**Affiliations:** 1 Department of Physics, Chongqing University of Arts and Sciences, Chongqing 402160, China; 2 Faculty of Mechanical and Electrical Engineering, Kunming University of Science and Technology, Kunming 650500, China; 3 Laboratoire de Physique Quantique de la Matiere et de Modelisation Mathematique (LPQ3M), Universite de Mascara, Mascara 29000, Algeria; khenata_rabah@yahoo.fr; 4 School of Physical Science and Technology, Southwest University, Chongqing 400715, China

**Keywords:** YB_2_, linear band crossing, topological metal, spin–orbit coupling, phonon dispersion, electronic structure

## Abstract

The presence of one-dimensional (1D) nodal lines, which are formed by band crossing points along a line in the momentum space of materials, is accompanied by several interesting features. However, in order to facilitate experimental detection of the band crossing point signatures, the materials must possess a large linear energy range around the band crossing points. In this work, we focused on a topological metal, YB_2_, with phase stability and a *P6/mmm* space group, and studied the phonon dispersion, electronic structure, and topological nodal line signatures via first principles. The computed results show that YB_2_ is a metallic material with one pair of closed nodal lines in the *k_z_* = 0 plane. Importantly, around the band crossing points, a large linear energy range in excess of 2 eV was observed, which was rarely reported in previous reports that focus on linear-crossing materials. Furthermore, YB_2_ has the following advantages: (1) An absence of a virtual frequency for phonon dispersion, (2) an obvious nontrivial surface state around the band crossing point, and (3) small spin–orbit coupling-induced gaps for the band crossing points.

## 1. Introduction

Since the discovery of topological insulators [1,2,3,4,5,6,7,8,9,10], the search for band topology has attracted considerable research interest. In recent years, nontrivial band topology has progressed from insulators to semimetals/metals [11,12,13,14,15,16,17,18,19,20,21,22,23,24,25]. Semimetals and metals that contain nontrivial band crossing points are known as topological semimetals and topological metals, respectively. The band crossings in topological semimetals and topological metals are not only limited to forming zero-dimensional band crossing points, they are also able to form nodal lines, i.e., one-dimensional (1D) band crossings, and nodal surfaces, i.e., two-dimensional (2D) band crossings, in the momentum space.

Nodal line materials [26,27,28,29,30,31,32,33,34,35] were first predicted in carbon-based networks and exhibit many interesting electronic and optical properties; furthermore, the associated nontrivial drum head-like surface states have emerged as an interesting research topic. Moreover, if multiple nodal lines conjoin, new types of topological materials are formed, such as nodal chain materials [36,37,38], nodal net materials [39], and nodal link materials [40,41,42].

However, following the discovery of new topological semimetals/metals, the detection of materials suitable for experimental operation represents a significant challenge. To be suitable for experimental operation, materials should have the following basic properties: (1) The target materials are already prepared or at the least are, in theory, phase stable, (2) a large linear energy range should be present around the band crossing points, and (3) the influence of spin–orbit coupling effect on the electronic structure should be relatively small [29].

In this study, we found that *P6/mmm*-type YB_2_ possesses the above-mentioned advantages. We would like to point out that *P6/mmm*-type YB_2_ is an existing material and its experimental lattice constants are a = b = 3.3042 Å, c = 3.8465 Å [43]. Note that Song et al. [44] revealed that high-purity YB_2_ powders can be obtained by vacuum solid-state reaction at 1800 °C for 10 h. We studied the structural stability, electronic structure, and topological signatures of this material via first principles. Our calculations showed that YB_2_ is a topological semimetal with one pair of nodal lines in the *k_z_* = 0 plane and a large linear band dispersion around the band crossings, which should facilitate further experimental investigation. To determine the structural stability of this system, the phonon dispersion was also examined. When the effect of spin–orbit coupling is considered, small gaps (up to 40 meV) are found at the band crossing points. Therefore, YB_2_ is a good candidate for the investigation of the physical properties of nodal line fermions.

## 2. Crystal Structure and Methods

**Figure 1:**
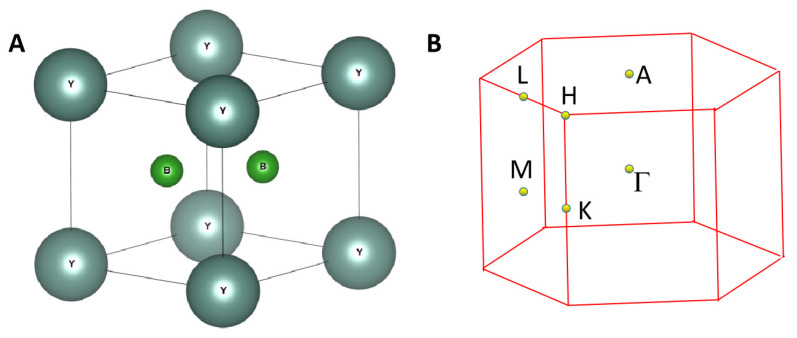
(**A**) Structural model of P6/mmm YB_2_, (**B**) the bulk Brillouin zone (BZ) of YB_2_.

Hexagonal-type YB_2_ with a *P6/mmm* space group was the focus of this study. According to the Materials Project Database [45], the formation energy per atom for YB_2_ is −0.564 eV and the Inorganic Crystal Structure Database IDs of 615708 and 44602 are assigned to YB_2_. The crystal structure of YB_2_ is shown in Figure 1A. One primitive cell contains one Y atom and two B atoms. The Y atom occupies the (0,0,0) sites and the two B atoms occupy the (0.3333, 0.6666, 0.5) and (0.6666, 0.3333, 0.5) sites.

We used first principles to calculate the electronic structure of the entire YB_2_ material. For the exchange-correlation potential, the generalized gradient approximation (GGA) [46] of the Perdew–Burke–Ernzerhof (PBE) [47] function was adopted. In this study, the cutoff energy was set at 600 eV and the Brillouin zone was sampled using the Monkhorst–Pack *k*-point mesh with a size of 11 × 11 × 8. To determine the nontrivial surface states in YB_2_, the WANNIERTOOLS package (version 2.5.0) [48] was selected.

The crystal structure of YB_2_ was fully relaxed and the optimized lattice parameters were found to be a = b = 3.29 Å and c = 3.85 Å. These values are in a good agreement with the experimental ones [43]. Also, these values match well those in the Materials Project Database (a = b = 3.30 Å, c = 3.85 Å).

**Figure 2:**
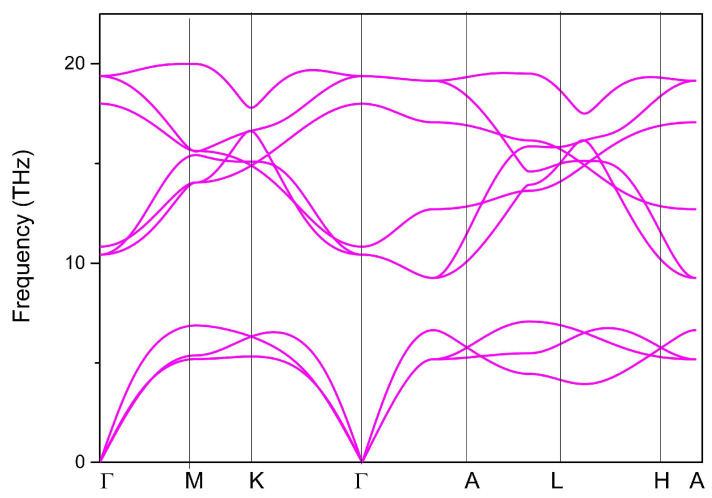
Phonon dispersion curve of YB_2_.

The Phonopy code was used to plot the phonon dispersion curve using the supercell-based approach. A 2 × 2 × 2 supercell was built and the phonon dispersion of the YB_2_ supercell along the Γ-M-K-Γ-A-L-H-A paths (see Figure 1B) is shown in Figure 2. The absence of imaginary frequencies in the first Brillouin zone confirms its dynamic stability.

## 3. Results and Discussion

**Figure 3:**
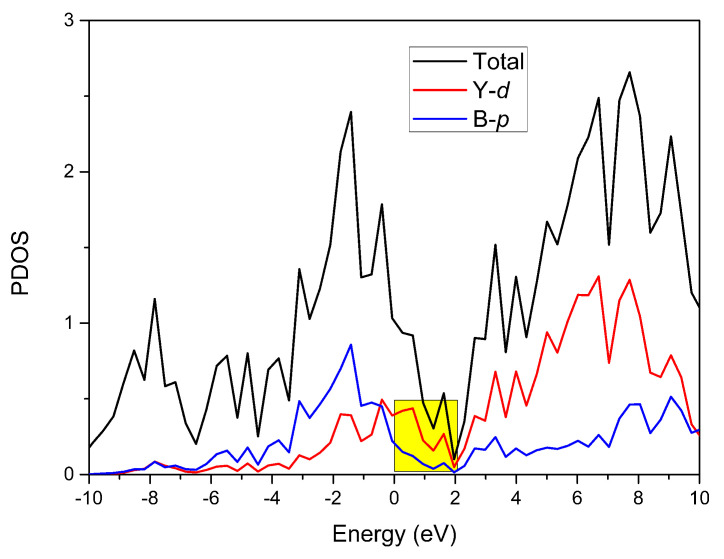
The total and projected density of states (PDOSs) of YB_2_ without considering the spin–orbit coupling effect. The Fermi level energy is set at zero.

Using the GGA–PBE method, the density of states of YB_2_ was produced and can be seen in Figure 3. We can see that this material exhibited metallic properties; however, the total density of states value was low, at around 2 eV. The total density of states in the range 0–2 eV was mainly dominated by Y-*d* orbitals (see the yellow area in Figure 3).

**Figure 4:**
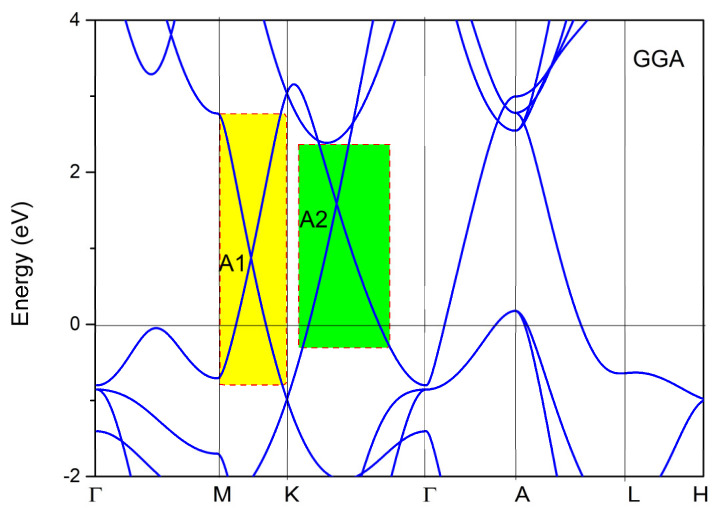
Calculated band structure of YB_2_ without considering spin-orbit coupling (SOC.) The Fermi level energy is set at zero.

**Figure 5:**
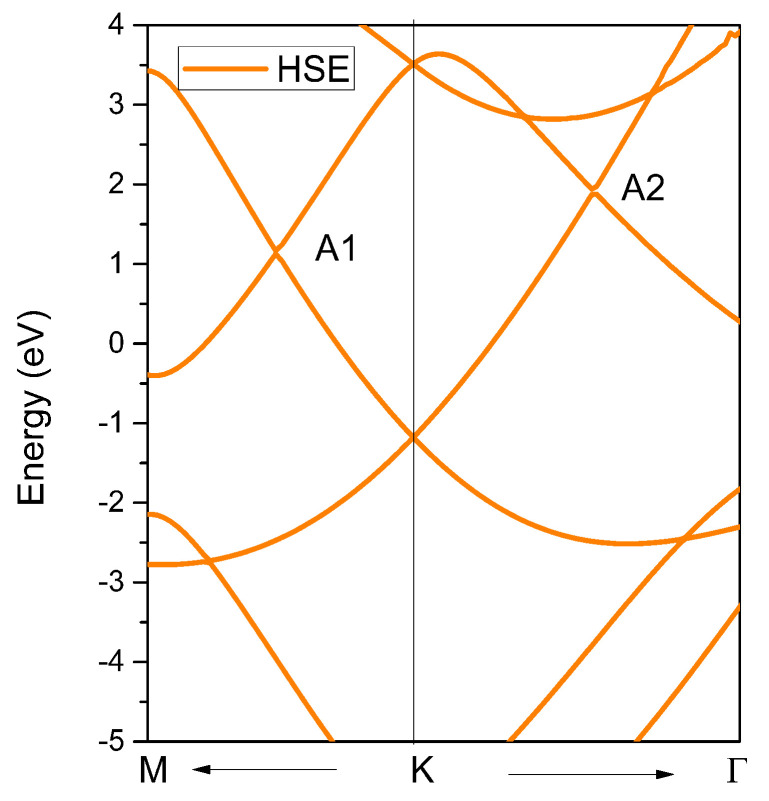
The band structure of YB_2_ calculated using Heyd−Scuseria−Ernzerhof (HSE) 06.

To fully understand the nontrivial band crossing points and the corresponding topological signatures, we also determined the band structure of YB_2_, as shown in Figure 4. From Figure 4, we can see two obvious band crossing points, named A1 and A2, near the K high symmetry points. Before discussing the topological signatures of both band crossing points, we should point out that the energy range of the linear band dispersion around the A1 and A2 band crossing points was more than 2 eV (see the yellow and green areas in Figure 4). Such a large linear energy range is substantially larger than most other proposed linear-type band dispersion materials [49]. Moreover, such a large linear energy range makes YB_2_ a promising candidate to study the physics related to experimental band crossings. To obtain an accurate range of the large linear band dispersion and band crossing points of YB_2_, the hybrid functional [50] was used to calculate the electronic structure along the M-K-Γ paths, and the results are shown in Figure 5. It is clear that the two band crossing points, A1 and A2, and the large range (larger than 2 eV) of the linear band dispersion were retained.

**Figure 6:**
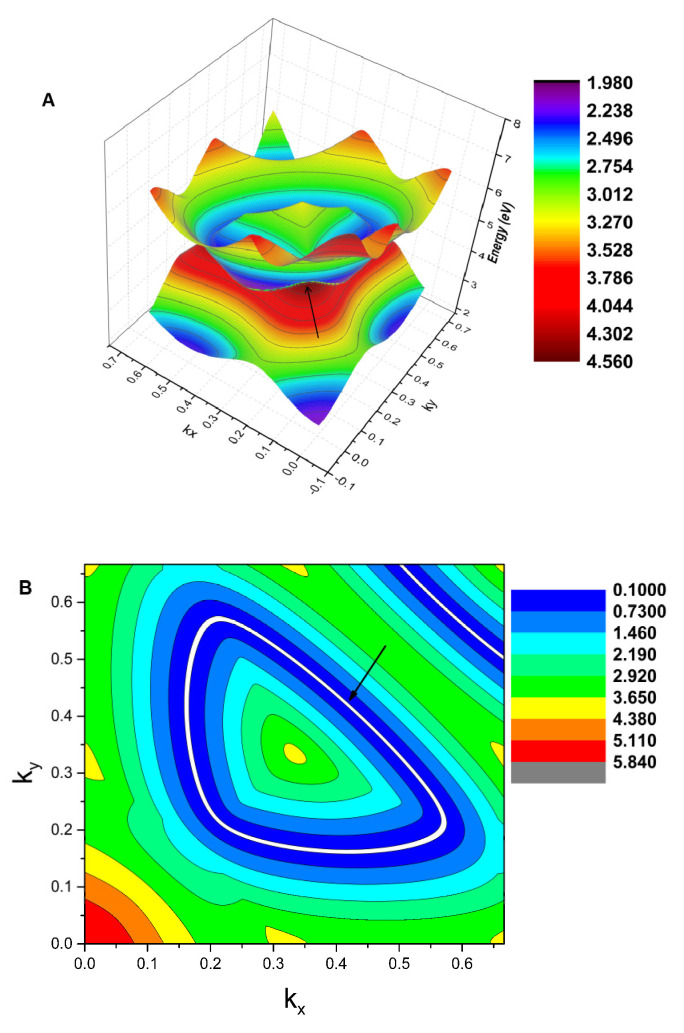
(**A**) The 3D band dispersion around the K point in the *k_z_* = 0 plane. The white line shows the profile of the nodal line. (**B**) The 2D shape of the K-centered nodal line in the *k_z_* = 0 plane (highlighted by the white line and indicated using an arrow).

Next, we discuss the topological signatures of these two band crossing points, A1 and A2, based on the arguments presented by Weng et al. [51]. These double-degenerated crossings should be assigned to a line, and the band crossing points should not be seen as isolated points. To further prove that A1 and A2 reside on a nodal line, the K-centered three-dimensional (3D) plot of the two bands in the *k_z_* = 0 plane as well as the K-centered 2D plot of the two bands in the *k_z_* = 0 plane are given in Figure 6A,B, respectively. The white lines in Figure 6 show the intersections between the two bands, namely, an obviously closed line. As shown in Figure 6A, we can see that the band crossing points belong to a nodal line in the *k_z_ =* 0 plane, and this nodal line has a slight energy variation. The 2D plane figure of the K-centered nodal line is shown in Figure 6B.

**Figure 7:**
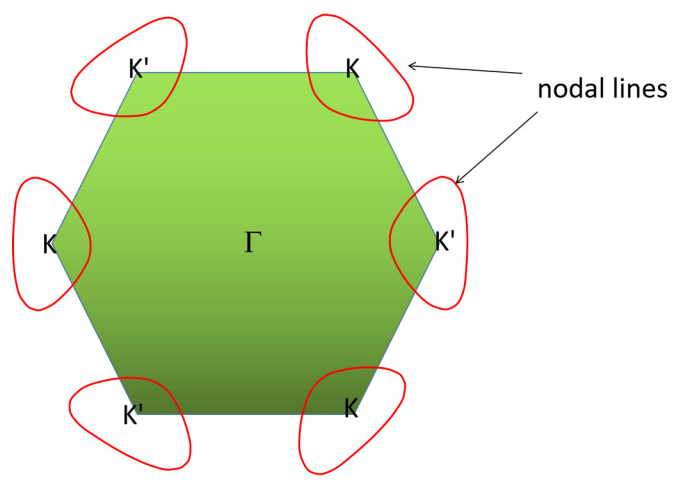
Illustration of one pair of nodal lines (highlighted by red lines) in the *k_z_* = 0 plane.

A YB_2_ crystal structure has two mechanisms to protect the nodal line: (1) A horizontal mirror plane with the nodal line located in the mirror-invariant *k_z_ =* 0 plane and (2) inversion symmetry and time-reversal symmetry. It should be noted that the system has time-reversal symmetry and, thus, there should be the same nodal line centered at the K’ point, as shown in Figure 7. Note that the band structures of ScB_2_, VB_2_, ZrB_2_, NbB_2_, HfB_2_, and TaB_2_ with *P6/mmm* structure were calculated by Zhang et al. [52]. Based on their work, one can see that these above-mentioned materials also have large linear energy range.

**Figure 8:**
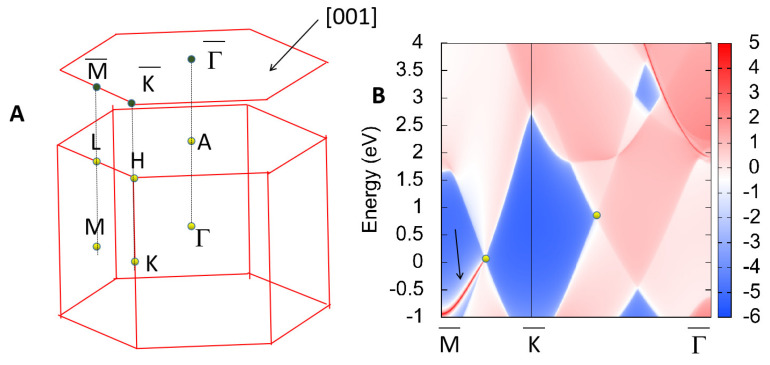
(**A**) The 3D bulk of BZ and the 2D surface of BZ, (**B**) the projected spectrum of the (001) surface of YB_2_. The band crossing points and the nontrivial surface states are highlighted, using yellow balls and red lines, respectively.

The surface states along the (001) direction for the nodal line are given in Figure 8. In Figure 8B, the band crossing points are shown using yellow balls and the nontrivial surface states using red lines. From Figure 8B, one can see that the surface states along the $\bar{K}-\bar{\Gamma }$ path are merged in the bulk state; however, the surface states along the $\bar{M}-\bar{K}$ path are clearly defined. Note that the investigation of nodal line material is at initial stage, and some of them [53,54,55] have been confirmed in experiment. For example, the nodal line fermions of ZrSiSe [55] were proven in de Haas–van Alphen (dHvA) quantum oscillations.

**Figure 9:**
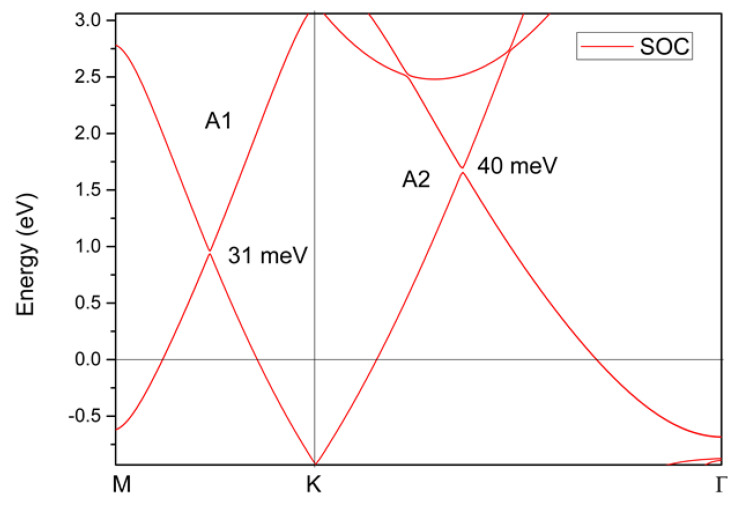
The band structure of YB_2_ along the M-K-Γ paths calculated using GGA. The spin–orbit coupling effect is taken into consideration.

The calculated electronic structures displayed above did not consider the spin–orbit coupling effect. Therefore, as a final consideration, we will discuss the effect of spin–orbit coupling on the band crossing points in the YB_2_ system. The results are shown in Figure 9; one can see that the spin–orbit coupling-induced gaps for points A1 and A2 were 31 meV and 40 meV, respectively. For almost all topological materials with nodal line states, gaps can be formed between their nodal lines via spin–orbit coupling effects. However, the spin–orbit coupling gaps in the YB_2_ system were relatively small in comparison to some well-known nodal line semimetals/metals, such as CaTe (~50 meV) [56], BaSn_2_ (>50 meV) [57], CaAgBi (>80 meV) [58], and TiOs (>100 meV) [59].

## 4. Conclusions

In conclusion, we have reported a perfect topological metal, *P6/mmm*-type YB_2_, using first principles. The *P6/mmm*-type YB_2_ has high structural stability, one pair of nodal lines in the *k_z_* = 0 plane, and a large linear energy range near the band crossings. The single pair of nodal lines are protected by two independent mechanisms: (1) Mirror symmetry and (2) inversion and time-reversal symmetries. We observed the nontrivial surface states in the (001) plane. Under the effect of spin–orbit coupling, gaps were present between the nodal lines with values of up to 40 meV. It should be noted that the spin–orbit coupling-induced gaps were smaller than some predicted nodal line state topological materials.
